# Use of Chinese Herbal Medicines Is Related to a Reduction in Depression Risk Among Patients With Insomnia: A Matched Cohort Study

**DOI:** 10.3389/fneur.2020.583485

**Published:** 2021-01-20

**Authors:** Yun-Wen Chiao, Hanoch Livneh, How-Ran Guo, Wei-Jen Chen, Ming-Chi Lu, Miao-Chiu Lin, Chia-Chou Yeh, Tzung-Yi Tsai

**Affiliations:** ^1^Department of Chinese Medicine, Dalin Tzuchi Hospital, The Buddhist Tzuchi Medical Foundation, Chiayi, Taiwan; ^2^Department of Traditional Medicine, Kaohsiung Veterans General Hospital, Kaohsiung, Taiwan; ^3^Rehabilitation Counseling Program, Portland State University, Portland, OR, United States; ^4^Department of Occupational and Environmental Medicine, National Cheng Kung University Hospital, Tainan, Taiwan; ^5^Occupational Safety, Health, and Medicine Research Center, National Cheng Kung University, Tainan, Taiwan; ^6^Department of Environmental and Occupational Health, College of Medicine, National Cheng Kung University, Tainan, Taiwan; ^7^Division of Allergy, Immunology and Rheumatology, Dalin Tzuchi Hospital, The Buddhist Tzuchi Medical Foundation, Chiayi, Taiwan; ^8^School of Medicine, Tzu Chi University, Hualien, Taiwan; ^9^Department of Nursing, Dalin Tzuchi Hospital, The Buddhist Tzuchi Medical Foundation, Chiayi, Taiwan; ^10^School of Post-Baccalaureate Chinese Medicine, Tzu Chi University, Hualien, Taiwan; ^11^Department of Medical Research, Dalin Tzuchi Hospital, The Buddhist Tzuchi Medical Foundation, Chiayi, Taiwan; ^12^Department of Nursing, Tzu Chi University of Science and Technology, Hualien, Taiwan

**Keywords:** insomnia, Chinese herbal medicine, depression, complementary and alternative, cohort study

## Abstract

**Objective:** Subjects with insomnia have a higher risk of depression, thus possibly making them live with serious health conditions. To date, information regarding the effect of Chinese herbal medicines (CHMs), a commonly used complementary and alternative medicine, on depression risk among people with insomnia is still unknown. This study aimed to investigate the effect of CHMs on the risk of depression among individuals with insomnia.

**Methods:** This cohort study used a national health insurance database to identify 68,573 subjects newly diagnosed with insomnia, aged 20–70 years, who received treatment between 1998 and 2010. Using propensity score matching, we randomly selected 26,743 CHMs users and 26,743 non-CHMs users from this sample. All enrollees were followed to the end of 2012 to identify any treatment for depression as the end point. Cox proportional hazards regression was used to compute the adjusted hazard ratio of depression associated with CHMs use.

**Results:** After utilizing the propensity score matching, we randomly selected 26,743 CHMs users and 26,743 non-CHMs users from this sample. During follow up, 3,328 CHMs users and 6,988 non-CHMs users developed depression at incidence rates of 17.24 and 37.97 per 1,000 person-years, respectively. CHMs users had a lower depression risk than the non-CHMs users (adjusted hazard ratio = 0.44; 95% Confidence Interval, 0.42–0.46). The greatest effect was observed for those taking CHMs for more than 2 years. Gegen, Huangqin, Dan-Shen, Beimu, Dahuang, Shegan, Shu-jing-huo-xue-tang, Ge-gen-tang, Shao-yao-gan-cao-tang and Píng wèi sǎn were significantly associated with a lower risk of depression.

**Conclusions:** Findings from this study demonstrated that adding CHMs to conventional treatment significantly reduces depression risk among patients with insomnia.

## Key Points

- This is the first investigation to explore the effects of CHMs use against the depression onset among individuals with insomnia.
- We discovered that the CHMs use was associated with a lower risk of depression after adjustment for the potential confounding factors.
- The more predominant effect was observed among those receiving CHMs for more than 2 years, with up to 80% lower risk of depression.
- In addition to the positive effect of CHMs, the findings of this study further indicated the commonly prescribed herbal products that may be related to reduced depression risk, thus paving the way for further pharmacological investigations.

## Introduction

Insomnia is a common sleep disorder affecting about one third of the general population worldwide. It is characterized by difficulty in falling asleep, difficulty in staying asleep, and sleep that is non-restorative in nature (1). Compared to the general population, those with persistent insomnia have a 58% increased risk of all-cause mortality (2). The rise in incidence of chronic insomnia not only triggers major health challenges but also results in tremendous economic loss. The total cost associated with insomnia is estimated at $92.5–107.5 billion annually in the US alone (3), which implied that insomnia was clearly an important public health concern requiring particular attention.

Notably, insomnia would further result in sleepiness, fatigue or cognitive impairment, thus pre-disposing individuals who experience insomnia to developing depression. A meta-analysis of 21 longitudinal studies demonstrated that insomnia may double the risk of depression independently of conventional risk factors (4). At its worst, a diagnosis of depression increases the risk of death by as much as 50% (5). Moved by the tragic case presented above, interventions geared at preventing or lessening the susceptibility of depression may be of utmost importance.

In Asian cultures, Chinese herbal medicines (CHMs) are complementary and alternative medical therapies commonly used for patients with chronic diseases. Several of these herbs have been proven to improve cognitive function by suppressing monoamine oxidase activity and oxidative stress, upregulating neurotrophins and modulating the function of the hypothalamic-pituitary-adrenal (HPA) axis in patients with neurodegenerative diseases (6). Previous research has illustrated that CHMs produce therapeutic effects of equal magnitude to hypnosis through multiple bioactivity channels in the central neural system (7, 8). One systematic review affirmed the efficacy and safety of CHMs to be as effective as that of antidepressants (9). Another pharmacological research summarized the antidepressant mechanisms of active components, such as elevated synaptic concentrations of monoamines, modifying the HPA axis dysfunctions, and increasing synaptic plasticity (10). Such therapeutic benefits bolster the potential of CHMs use for treating depression in patients with insomnia.

Considering the potential association of insomnia with a subsequent risk of depression and the limited information on the capacity of CHMs to modify this association, we believe findings from a long-term population-based cohort study may be of importance in allocating medical resources and instituting evidence-based policymaking. Thus, we examined a nationwide population-based database to compare the risk of depression among patients with insomnia who did and did not receive CHMs.

## Methods

### Data Source

The present cohort study analyzed data from the Longitudinal Health Insurance Database (LHID 2000), which is managed by the National Health Insurance (NHI) Administration. Taiwan launched a single-payer NHI program in 1995 to remove financial barriers to medical care for all legal residents. As of 2017, over 99% of Taiwan's population had enrolled in this program. The LHID 2000 is based on a sub-set of the NHI program made up of one million randomly sampled people who were traced retrospectively from 1996 to 2012. These one million insured individuals did not differ significantly in sex or age from the general population, as a multistage stratified systematic sampling method was used (11). This database contains all NHI enrollment files, claims data and data from a prescription drugs registry, providing comprehensive utilization information on the subjects covered by the insurance program.

### Ethics

The current investigation used retrospective data from an administrative database in which patients were made anonymous to the researchers. It was also conducted in accordance with the Helsinki Declaration and was approved by the Institutional Review Board and Ethics Committee of Buddhist Dalin Tzu Chi Hospital, Taiwan (No. B10004021-3).

### Study Participants

**Figure 1:**
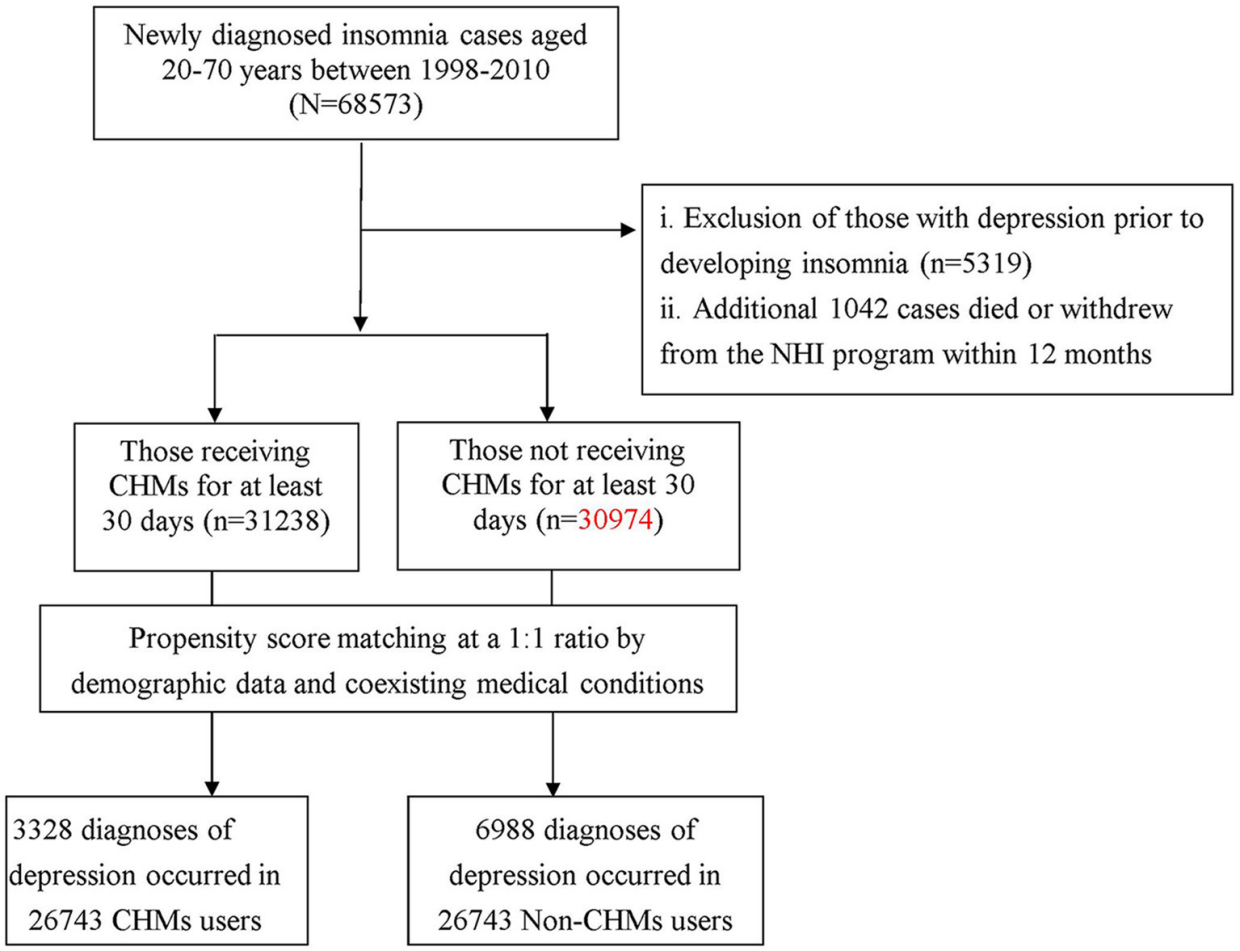
Flow chart of selection and follow-up of study subjects.

The methods used to select the study subjects are illustrated in Figure 1. Diagnoses in the insurance claims data were coded using the International Classification of Disease, 9th Revision, Clinical Modification (ICD-9-CM). Patients who were older than 20 years, and were newly diagnosed with insomnia between 1998 and 2010 (ICD-9-CM codes: 307.41, 307.42, 780.50, 780.52), were considered for enrollment into the study.

To reduce the potential for disease misclassification, we selected patients had diagnoses of insomnia in at least three outpatient visits, or were admitted to the hospital with a primary diagnosis of insomnia within the observation period (*n* = 68,573). To establish a temporal link between insomnia and depression, we excluded those who were followed for <1 year after the onset of insomnia (*n* = 1,042) and those who had a prior diagnosis of depression before the first insomnia diagnosis (*n* = 5,319). Patients were identified as having depression if they had at least three ambulatory visits for treatment or had been hospitalized due to depression, as reflected in the use of ICD-9-CM codes 296.2, 296.3, 300.4, or 311. A group of 62,212 subjects with insomnia were included in the final data analysis.

As only certified Chinese medicine physicians are permitted to prescribe CHMs in Taiwan. We, therefore, used the frequency of visits to Chinese medicine physicians to verify the CHMs exposure of each insomniac patient. Those receiving CHMs to treat insomnia for more than 30 days were deemed CHMs users, whereas those treated for 30 days or less were considered non-CHMs users (12, 13). Based on this procedure, 31,238 cases were designated as CHMs users. A comparison cohort was randomly selected from the remaining insured insomnia cases without CHMs use. For each patient receiving CHMs treatment, one control without CHMs was selected by 1:1 matching based on a propensity score. The propensity score was calculated using logistic regression based on patient demographics and baseline comorbidities at enrollment. To consider the immortal time bias (14), we defined the index date for the follow-up period for the patients with insomnia who were classified as non-CHMs users to be the date of the first insomnia diagnosis. The index date for the follow-up period for the patients with insomnia who used CHMs was the first date of the initiation of CHMs treatment. Each patient with insomnia was followed from the index date until 31 December 2012, death or being censored. The follow-up time, in person-years (PYs), was calculated for each insomniac patient until the diagnosis of depression, death or until being censored due to withdrawal from the insurance system or loss to follow-up.

### Covariates Assessment

Covariates consisted of the baseline sociodemographic characteristics and comorbidities. Sociodemographic characteristics considered in this study included age, gender, income (for estimating insurance payments) and urbanization level of the registered resident region. Monthly income was defined as the subject's own insurable wage and stratified into three levels: ≤17,880 New Taiwan Dollar (NTD) (low); 17,881–43,900 NTD (median); and ≥43,901 NTD (high). In addition, we also grouped the resident region by collapsing each geographic location into one of three strata, namely, urban (levels 1–2), suburban (levels 3–4) and rural (levels 5–7), with higher levels indicating greater urbanization (15). Baseline comorbidities for each subject were determined by individual medical records in the year preceding cohort entry, all of which were assessed by the established Charlson-Deyo comorbidity index (CCI) (16). The CCI score consisted of 17 chronic diseases, each with a score of 1-to-6 points. The sum of these scores was regarded as a continuous variable for the burden of comorbidities, with higher scores indicating a more severe impact from the these comorbidities.

### Statistical Analysis

The SAS version 9.3 software (SAS Institute Inc, Cary, NC, USA) was used for data analysis. Distributions of socio-demographic data and comorbidities between the enrollees who did and did not receive CHMs were compared by the chi-square test and independent Student's *t*-test. Cox proportional hazards regression analysis was applied to compute the adjusted hazard ratio (HR) with its 95% confidence interval (CI) of the risk of depression in association with CHMs use, after accounting for the confounders reported at baseline. To further test the robustness of the relationship between CHMs use and depression risk, we divided the CHMs users into three subgroups: those using CHMs for 31–365 days, for 366–730 days and for more than 730 days. The Cox proportional hazards regression analysis with Bonferroni correction was applied for multiple comparisons among the groups. We also used the Kaplan-Meier method to estimate the cumulative risk of depression for different groups and tested the difference with the log-rank test. Furthermore, a stratified analysis by age and sex using Cox proportional hazards regression was conducted to assess the relative risk of depression associated with CHMs. The proportional hazards assumption was examined by plotting the log[-log(survival function)] vs. the log of survival time. Differences of *p* < 0.05 were determined to be statistically significant.

## Results

The CHMs user and non-CHMs user cohorts each provided data for 26,743 patients. Table 1 shows the pertinent characteristics of the two groups, including the distribution of age, sex, monthly income, residential area and comorbidities, and indicating that the two groups were comparable on these characteristics. The mean age of the CHMs users and non-CHMs users was 48.5 ± 12.3 and 48.5 ± 12.5 years, respectively. Female patients predominated in both groups, at 64.0%. The majority of participants had monthly income levels of 17,881–43,900 NTD (48.7%) and resided in more urbanized areas (57.8%).

**Table 1:** Demographic data and selected comorbidities of the study subjects.

**Variables**	**All**	**Non-CHMs users**	**CHMs users**	*p-*value^*^
		***n* = 26,743(%)**	***n* = 26,743(%)**	
Age (yr)				0.44
≤50	28,771 (53.8)	14,341 (53.6)	14,430 (54.0)	
>50	24,715 (46.2)	12,402 (46.4)	12,313 (46.0)	
Mean (SD)	48.5 ± 12.4	48.5 ± 12.3	48.5 ± 12.5	0.97
Gender				0.69
Female	34,224 (64.0)	17,090 (63.9)	17,134 (64.1)	
Male	19,262 (36.0)	9,653 (36.1)	9,609 (35.9)	
Monthly income				0.40
Low	24,988 (46.7)	12,417 (46.4)	12,571 (47.0)	
Median	26,037 (48.7)	13,095 (49.0)	12,942 (48.4)	
High	2,461 (4.6)	1,231 (4.6)	1,230 (4.6)	
Residential area				0.96
Urban	30,911 (57.8)	15,454 (57.8)	15,457 (57.8)	
Suburban	8,420 (15.7)	4,221 (15.8)	4,199 (15.7)	
Rural	14,155 (26.5)	7,068 (26.4)	7,087 (26.5)	
CCI				0.28
Mean (SD)	4.0 (6.7)	3.9 (6.6)	4.0 (6.8)	

*CHMs, Chinese herbal medicines; SD: standard deviation; CCI, Charlson-Deyo comorbidity index*.

* *All p-values were derived from chi-square test, except for CCI that was calculated with independent Student's t-test*.

**Figure 2:**
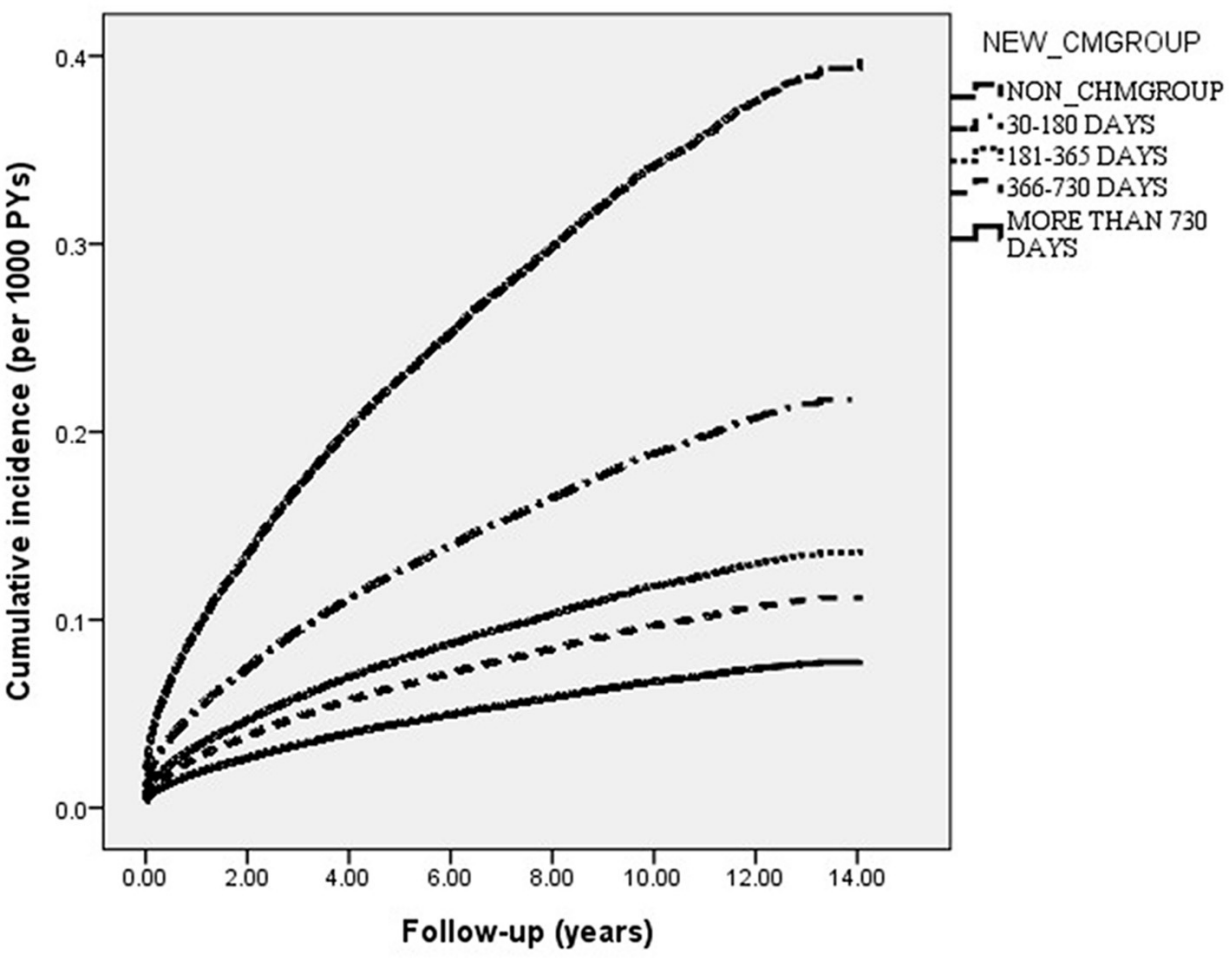
Cumulative incidence of depression in insomnia patients with and without receiving CHMs treatment during the 15-year study period (Log-rank test, *P* < 0.001).

Among the 53,486 patients with insomnia and no history of prior depression, who were enrolled in this study, 10,316 first episodes of depression were identified; 6,988 were reported among the non-CHMs users and 3,328 among the CHMs users during follow-up periods of 184,029.27 and 193,037.73 PYs, respectively. The incidence rate of depression was lower among CHMs users than in non-CHMs users (17.24 vs. 37.97, respectively, per 1000 PYs), with the adjusted HR of 0.44 (95% CI = 0.42–0.46) (Table 2). Table 2 also lists the crude and adjusted HRs with Bonferroni correction using the non-CHMs users as reference. Those who used CHMs treatment for more than 730 days had an 80% lower risk of depression than non-CHMs users (adjusted HR = 0.20; 95% CI = 0.18–0.23). Figure 2 presents the Kaplan–Meier estimates of the cumulative incidence of depression for the four groups during the 15-year follow-up period, after adjusting for patients' age, sex, CCI scores, monthly incomes and urbanization levels. The cumulative incidence of depression of those receiving CHMs treatments for more than 730 days was significantly lower than for those not receiving CHMs (log-rank test, *p* < 0.001).

**Table 2:** Risk of depression for insomnia subjects with and without CHMs.

**Patient group**	**Event**	**PYs**	**Incidence (per 1000 PYs)**	**Crude HR (95% CI)**	Adjusted HR^*^ (95% CI)
Non-CHMs users	6,988	184029.27	37.97	1	1
CHMs users	3,328	193037.73	17.24	0.45 (0.43–0.47)	0.44 (0.42–0.46)
CHMs use within 30–180 days	2,352	107147.47	21.95	0.58 (0.53–0.60)	0.55 (0.53–0.58)
CHMs use within 181–365 days	527	38744.11	13.60	0.36 (0.33–0.39)	0.34 (0.31–0.37)
CHMs use within 366 to 730 days	311	28278.19	11.00	0.29 (0.26–0.33)	0.28 (0.25–0.31)
CHMs use for more than 730 days	138	18867.96	7.31	0.20 (0.17–0.23)	0.20 (0.18–0.23)

* *Model adjusted for age, gender, urbanization level, monthly income, medication usage and CCI score*.

We performed an additional stratified analysis by age and sex to determine the effect of CHMs on risk of depression (Table 3). Findings showed that both female and male patients with insomnia, who also received CHMs, had a lower risk of depression than did their non-CHMs counterparts, with an adjusted HR of 0.41 (95% CI = 0.38–0.43) and 0.53 (95% CI = 0.49–0.57), respectively. A more remarkable beneficial effect of CHMs was observed among younger subjects. In females, decreases in adjusted HR were greater for CHMs users ≤50 years of age (adjusted HR = 0.32, 95% CI = 0.31–0.35); in males, the effect of CHMs on the risk of depression was greater for those ≤50 years of age, with an adjusted HR of 0.45 (95% CI = 0.41–0.50).

**Table 3:** Incidence (per 1000 PYs) and depression risk for insomnia patients with and without CHMs in the stratification of sex and age.

**Variables**	**Non-CHMs users**			**CHMs users**			**Crude HR (95% CI)**	**Adjusted HR (95% CI)**
	**Case**	**PYs**	**Incidence**	**Case**	**PYs**	**Incidence**		
Female								
≤50	2,949	52986.41	55.66	1,158	64092.24	18.07	0.32 (0.30–0.35)	0.32 (0.31–0.35)^*^
>50	2,026	60012.79	33.76	1,106	59922.36	18.46	0.55 (0.49–0.57)	0.53 (0.50–0.57)^*^
All	4,975	112999.2	44.03	2,264	124014.6	18.26	0.41 (0.42–0.46)	0.41 (0.38–0.43)^Ω^
Male								
≤50	1,296	39882.51	32.50	590	39102.37	15.09	0.46 (0.41–0.50)	0.45 (0.41–0.50)^*^
>50	717	31207.56	22.98	474	29920.76	15.84	0.69 (0.59–0.75)	0.66 (0.59–0.74)^*^
All	2,013	71090.07	28.32	1,064	69023.13	15.42	0.54 (0.49–0.57)	0.53 (0.49–0.57)^Ω^

* *Model adjusted for urbanization level, monthly income, medication usage and CCI score*.

Ω *Model adjusted for age, urbanization level, monthly income, medication usage and CCI score*.

The most commonly prescribed Chinese herbal products for insomnia patients are summarized in Table 4. Of these, 10 were found to substantially lessen the subsequent risk of depression: Ge Gen (*Pueraria lobate*), Huang Qin (*Scutellaria Baicale*), Dan Shen (*Salvia miltiorrhiza*), Bei Mu (*Fritillariae Thunbergii*), Da Huang (*Rheum palmatum*), She Gan (*Belamcanda chinensis*), Shu-jing-huo-xue-tang (SJHXT), Ge-gen-tang (GGT), Shao-yao-gan-cao-tang (SYGCT) and Ping-wei–san (PWS).

**Table 4:** Risk of depression in relation to the 10 most-used single-herb and multi-herb products for insomnia patients.

**Chinese herbal name**	**Number of prescriptions**	**Crude HR (95% CI)**	Adjusted HR ^*^ (95% CI)
**Single-herb products**			
Yan-Hu-Suo	11,868	0.81 (0.68–1.09)	0.84 (0.69–1.11)
Gegen	11,549	0.46 (0.43–0.49)	0.44 (0.42–0.46)
Huangqin	10,291	0.46 (0.44–0.49)	0.44 (0.42–0.47)
Multiflora	9,803	0.86 (0.72–1.10)	0.88 (0.73–1.09)
Dan-Shen	9,234	0.47 (0.44–0.46)	0.46 (0.43–0.49)
Beimu	9,116	0.43 (0.41–0.46)	0.42 (0.39–0.45)
Dahuang	6,748	0.49 (0.45–0.52)	0.45 (0.42–0.48)
Suan Zao Ren	6,379	0.87 (0.73–1.06)	0.85 (0.73–1.11)
Hai Piao Xiao	6,051	0.85 (0.64–1.08)	0.84 (0.63–1.09)
Shegan	4,527	0.44 (0.40–0.48)	0.42 (0.38–0.46)
**Multi-herb products**			
Jia-Wei-Xiao-Yao-San	14,573	0.89 (0.73–1.09)	0.88 (0.74–1.05)
Shu-Jing-Huo-Xue-Tang	12,684	0.45 (0.42–0.47)	0.45 (0.42–0.48)
Ge-Gen-Tang	11,549	0.46 (0.43–0.49)	0.45 (0.42–0.48)
Shao-Yao-Gan-Cao-Tang	11,212	0.43 (0.41–0.46)	0.43 (0.40–0.45)
Chuan Xiong Cha Tiao San	9,162	0.87 (0.75–1.07)	0.85 (0.72–1.06)
Píng wèi sǎn	7,931	0.46 (0.43–0.49)	0.45 (0.42–0.47)
Suan Zao Ren Tang	7,883	0.86 (0.72–1.08)	0.89 (0.74–1.07)
Tian Wang Bu Xin Dan	7,706	0.88 (0.74–1.07)	0.89 (0.74–1.10)
Ban Xia Xie Xin Tang	7,566	0.89 (0.76–1.08)	0.88 (0.74–1.11)
Xiao Chai Hu Tang	6,752	0.89 (0.78–1.13)	0.89 (0.76–1.14)

* *Model adjusted for age, sex, urbanization level, monthly income, and comorbidities*.

## Discussion

This is the first large scale, retrospective cohort study exploring the risk of depression in patients with insomnia who either received or did not receive CHMs treatment. Although several studies were reported on the benefit of using CHMs for sleep disorders, or for other psychological problems (17–19), no studies have yet explored the relationship of CHMs with the risk of depression among those with sleep disorders.

The findings of the present study are consistent with those of prior epidemiological studies, showing that insomnia usually occurs before the first instance of depression (20). Our study lends initial support for integrating CHMs into conventional therapy, and suggests that such practice may reduce the risk of depression by as much as 60%. The dose-response relationship reported herein could further clarify the causality between CHMs use and a reduced susceptibility to develop depression. Our results indicate that the extracts of some Chinese herbal products may suppress the production of inflammatory cytokines and modulate the neurotransmitter imbalance that results in depression.

For example, the commonly prescribed multi-herb formula SJHXT was found to efficiently diminish the risk of depression in patients with insomnia. Using a rodent model, Shu and colleagues discovered that SJHXT could modulate the activity of the alpha-2 adrenoceptor (α2-AR) (21). A subsequent study indicated that depressive disorders appeared to be associated with increased α2AR sensitivity and responsiveness (22). The dysregulation of the α2-AR pathway may contribute to the release of pro-inflammatory cytokines, such as tumor necrosis factor-α (TNF-α), interleukin-1 (IL-1) and IL-6, thus increasing the susceptibility to depression (23).

The current study indicated that the use of SYGCT could lessen the risk of depression for those with insomnia. We speculate that the conspicuous anti-depressant effect of SYGCT may be related to its ingredients, including *Paeonia lactiflora* Pall. and *Glycyrrhiza uralensis*. A recent study showed that *P. lactiflora* Pall., a major compound purified from this formula, can significantly inhibit the hyperfunction of the HPA axis (24). Meanwhile, *G. uralensis* works by promoting the release of 5-hydroxytryptamine and increasing synaptic plasticity in the hippocampus (10), thereby allowing neurotransmitters to function more efficiently.

Another herbal product proven effective in lessening depression risk is PWS. An earlier report suggests that *Magnolia officinalis*, a major component in PWS, can inhibit neuroinflammation and oxidative stress in the prefrontal cortex and improve the levels of brain-derived neurotrophic factor (BDNF) protein in the hippocampus, as shown in a rodent model of depressive disorder (25). We also identified the positive therapeutic effects of GGT, as well as of Ge Gen. We speculate that puerarin, a major isoflavone glycoside purified from these agents, may increase cerebral blood flow via multiple neuroprotective effects, including anti-apoptosis and anti-inflammatory effects, and reduce oxidative stress (26), which may point to the possible mechanisms responsible for the positive effects of these formulae.

With respect to other commonly used single-herb products, we observed that the use of Dan-Shen was related to a reduced risk of depression onset. This remedy has been shown to protect mice from Aβ-induced neurotoxicity by inhibiting increases in TNF-α, IL-6 or TNF-α levels (27, 28). These mediators are well-known to play important roles in the pathogenesis of neuropsychiatric symptoms, especially depression (29).

Additionally, Huang-Qin, Da-Huang, Bei-Mu and She-Gan are believed to exert their neuroprotection effect by suppressing pivotal activities of nuclear factor kappa-light-chain-enhancer of activated B cells associated with inflammatory pathways and cell apoptosis (30–34). Furthermore, the baicalin in Huang-Qin and emodin in Da-Huang can mediate BDNF expression, ameliorating depressive-like behavior (35, 36). BDNF in brain areas related to emotions, such as the hippocampus and cortex, can support the survival of neurons, as well as the differentiation and growth of new synapses (37, 38). Finally, it has been argued that tectorigenin, an isoflavonoid found in She-Gan, has a confirmed neuroprotective effect against cytotoxicity and apoptosis (39, 40).

Findings regarding the positive therapeutic effect of herbal formulas observed in this study, however, were not compatible with the findings from an earlier report (41). It could be speculated that these conflicting findings are partially due to the differences in the classifications of exposure of interest. For example, in the study of Chen and colleagues, enrollees were recruited with a single diagnostic code for insomnia, which differed from the approach we adopted. Priori study had pointed out while using the administration database, the algorithm requiring at least 3 outpatient service claims, or at least 1 inpatient hospitalization claim during the study period (42). The adopted procedure greatly strengthened the diagnostic validity and minimized possible potential misclassifications of our study.

While our study is the first to investigate the relation between CHMs use and depression risk among patients with insomnia, there are several important limitations. First, when using secondary health care databases, there is always the risk of errors in the coding process. To minimize this bias, we enrolled only persons with new-onset insomnia or depression, and only after the patients had at least three outpatient visits reporting a consistent diagnoses or at least one such inpatient admission. It should also be noted that the NHI of Taiwan randomly reviews the charts and audits medical charges to verify the accuracy of claims. However, the coding approach and data availability were similar between the two groups, and any misclassification bias would have likely been non-differential and toward the null hypothesis, thus, if anything, resulting in an underestimation of the observed effect. Second, the LHID lacks information on social network relationships, family history, personality attributes, laboratory data and education level. Thus, future studies that include these factors would be beneficial in shedding further light on our findings. Third, although continuous use of CHMs in our study indicated a significant benefit, well-designed randomized controlled trials are still needed to confirm the effect of specific medicines. These limitations notwithstanding, this study also had considerable advantages. The database used in this study is representative of the entire Taiwanese population, and the large sample size ensured reliable findings. In addition, the use of a longitudinal cohort study design allowed the robust examination of the causal relationship between high intensity CHMs use and lower risk of depression in people with insomnia.

## Conclusion

This is the first large population-based investigation to delineate the impact of CHMs on the risk of depression among patients with insomnia. The findings could serve as references for further studies concerning the influence of CHMs on other medical complications resulting from insomnia. We found that the integration of CHMs into conventional therapy reduced the subsequent risk of depression by 56%, with greater benefits in those receiving CHMs for longer than 2 years. Healthcare providers may consider integrating CHMs into therapeutic care to ameliorate the clinical manifestations of insomnia in their patients.

## Data Availability Statement

The raw data supporting the conclusions of this article will be made available by the authors, without undue reservation.

## Ethics Statement

The current study involving human participants was reviewed and approved by the Institutional Review Board and Ethics Committee of Buddhist Dalin Tzu Chi Hospital, Taiwan (No. B10004021-3). The ethics committee waived the requirement of written informed consent for participation.

## Author Contributions

Y-WC, C-CY, W-JC, and T-YT: study concept and design. M-CLu, T-YT, and H-RG: acquisition of data. T-YT, HL, and H-RG: data analysis. M-CLu and C-CY: project management. M-CLi, HL, T-YT, Y-WC, H-RG, and W-JC: writing. All authors contributed to the article and approved the submitted version.

## Conflict of Interest

The authors declare that the research was conducted in the absence of any commercial or financial relationships that could be construed as a potential conflict of interest.
